# Distinct expression profile of HCMV encoded miRNAs in plasma from oral lichen planus patients

**DOI:** 10.1186/s12967-017-1222-8

**Published:** 2017-06-07

**Authors:** Meng Ding, Xiang Wang, Cheng Wang, Xiaoshuang Liu, Ke Zen, Wenmei Wang, Chen-Yu Zhang, Chunni Zhang

**Affiliations:** 1 Department of Clinical Laboratory, Jinling Hospital, State Key Laboratory of Analytical Chemistry for Life Science, Advance Research Institute of Life Sciences, Nanjing University School of Life Sciences, Nanjing University, Nanjing, 210002 China; 2 State Key Laboratory of Pharmaceutical Biotechnology, Jiangsu Engineering Research Center for MicroRNA Biology and Biotechnology, Advance Research Institute of Life Sciences, Nanjing University School of Life Sciences, Nanjing University, Nanjing, 210046 China; 3 Department of Oral Medicine, Nanjing Stomat-ological Hospital, Medical School of Nanjing University, Nanjing, 210000 China

**Keywords:** Cytomegalovirus, MicroRNAs, Oral lichen planus, Cytomegalovirus UL16-binding protein 1, Plasma

## Abstract

**Background:**

Oral lichen planus (OLP) is a T cell-mediated autoimmune disease. The aetiology and molecular mechanisms of OLP remain unclear. Human cytomegalovirus (HCMV) infection is a causal factor in the development of various diseases, but the clinical relevance of HCMV in OLP has not been thoroughly investigated.

**Methods:**

In the present study, we firstly examined twenty-three HCMV-encoded microRNA (miRNA) expression profiles in plasma from training set that including 21 OLP patients and 18 healthy controls using RT-qPCR technology. Dysregulated miRNAs were subsequently confirmed in another larger cohort refereed as validation set consisting of 40 OLP patients and 33 healthy controls. HCMV DNA in peripheral blood leukocytes (PBLs) was also measured in an additional cohort of 13 OLP patients and 12 control subjects. Furthermore, bioinformatics analyses, luciferase reporter assay and western blotting were also performed to predict and verify the direct potential targets of HCMV-encoded miRNAs.

**Results:**

The RT-qPCR results showed that the plasma levels of five HCMV-encoded miRNAs including hcmv-miR-UL112-3p, hcmv-miR-UL22a-5p, hcmv-miR-UL148d, hcmv-miR-UL36-5p and hcmv-miR-UL59 were significantly increased in OLP patients in both training and validation sets. HCMV DNA in PBLs was also significantly higher in OLP patients than in control subjects. Additionally, by using a combination of luciferase reporter assay and western blotting, we demonstrated that cytomegalovirus UL16-binding protein 1, a molecule that mediates the killing of virus-infected cells by natural killer cells, is a direct target of hcmv-miR-UL59.

**Conclusions:**

Our results demonstrate a distinct expression pattern of HCMV-encoded miRNAs in OLP patients, which may provide insight into the relationship between HCMV infection and OLP, and warrants additional study in the diagnosis and aetiology of OLP.

**Electronic supplementary material:**

The online version of this article (doi:10.1186/s12967-017-1222-8) contains supplementary material, which is available to authorized users.

## Background

Oral lichen planus (OLP) is a relatively common chronic inflammatory disease of the oral mucosa that affects 0.1–4.0% of the adult population, with middle-aged and aged females exhibiting higher incidences [1, 2]. OLP affects oral mucous membranes with a variety of clinical presentations, including reticular, erosive, plaque-like, atrophic and ulcerative lesions [3], and the reticular and erosive type are most common clinically. Various therapeutic regimens have been designed to treat OLP. However, the highly relapsing nature of OLP is a great clinical challenge to improve the treatment result of the disease. Moreover, OLP is classified as a precancerous condition by the World Health Organization (WHO) [4]. Opinions on the pathogenesis of OLP vary, but it is currently accepted that OLP is a T-cell-mediated autoimmune disease [5]. However, the aetiology and molecular mechanisms underlying this autoimmune disease remain unclear.

MicroRNAs (miRNAs) are a subset of non-coding RNA molecules (21–23 nucleotides in length) that mediate post-transcriptional gene silencing [6]. MiRNAs are involved in various physiological and pathological processes, such as inflammatory and immune responses [7], in addition to their remarkable regulatory roles in the initiation and progression of human malignancies [8, 9]. Mounting discoveries demonstrated that serum or plasma contain a large amount of stable miRNAs that are derived from the host itself and other species, such as viruses, that infect the host, and certain dysregulated miRNAs in circulation are involved in the pathological processes of human diseases [10–12].

Human cytomegalovirus (HCMV) is a member of the herpesviridae family,betaherpesvirinae subfamily that latently infects approximately 70–100% of the population worldwide for their lifetime [13]. HCMV infection is generally asymptomatic in immunocompetent individuals, but it is also implicated in cardiovascular disease, arthritis and aggressive brain tumours [14, 15]. Since the report in 2004 that virus express miRNAs [12], at least 26 HCMV-encoded mature miRNAs have been discovered [16]. Some HCMV-associated miRNAs are related to viral proliferation, latency and immunoevasion [17]. For example, hcmv-miR-UL112-3p is highly expressed in plasma of hypertensive patients and associated with an increased risk of hypertension [18]. Hcmv-miR-UL112-3p targets IRF-1 and MICB, which are two key molecules in immunological, inflammatory and anti-infective responses, and IRF-1 and MICB repression by hcmv-miR-UL112-3p may be a unifying mechanism that evades the host response in the pathogenesis of hypertension [18]. A higher prevalence of hcmv-miR-UL112-3p was also detected in patients with diabetes mellitus and glioblastoma multiforme [19]. Hcmv-miR-US4-1 also targets ERAP1b mRNA during viral infection, which resulted in the inhibition of cytotoxic T lymphocyte (CTL) immune responses [20]. Our previous study showed that serum hcmv-miR-US4-1 can serve as a novel biomarker for predicting the outcome of interferon α treatment in chronic hepatitis B patients [21].

The clinical relevance and significance of HCMV in OLP have not been thoroughly investigated. This study examined HCMV-encoded miRNA expression profile in the plasma from patients with OLP. We found that the plasma expression pattern of HCMV-encoded miRNAs in patients with OLP was different from normal controls, and 5 of 23 studied HCMV miRNAs were upregulated in OLP patients. HCMV DNA in peripheral blood leukocytes (PBLs) was also significantly higher in OLP patients than in control subjects. While the positive rate of anti-HCMV IgG and anti-HCMV IgM were not different between OLP patients and normal control, which is consistent with the previous report [22]. Furthermore, we demonstrated that CMV UL16-binding protein 1 (ULBP1), which is a molecule that mediates the killing of virus-infected cells by natural killer (NK) cells, is a direct target of hcmv-miR-UL59. Together, our results demonstrate a set of upregulated HCMV-encoded miRNAs in OLP patients, which warrants additional study in the diagnosis and aetiology of OLP.

## Methods

### Patients, sample collection and RNA isolation

The present study enrolled 95 OLP patients, all of whom were newly diagnosed and have not yet been treated at Nanjing Stomatological Hospital. In addition, the recruitment of 63 subjects to the parallel control group was conducted in the Healthy Physical Examination Centre of Jinling Hospital. The health checkup included a detailed history, physical and ultrasonographic examinations and blood tests. The detailed clinical and grouped information can be seen in Table 1. All blood samples were collected in EDTA tubes using a standard operating procedure, and centrifuged and stored as previously described [23]. Total RNA was extracted from 100 μL plasma using a 1-step phenol/chloroform purification protocol as previously described [24]. In brief, 100 μL plasma was mixed with 300 μL deionized water, 200 μL acid phenol, and 200 μL chloroform. The mixture was vortex-mixed vigorously and incubated at room temperature for 15 min. After phase separation, the aqueous layer was mixed with 1.5 volumes of isopropyl alcohol and 0.1 volumes of 3 mol/L sodium acetate (pH 5.3). This solution was stored at −20 °C for 1 h. The RNA pellet was collected by centrifugation at 16,000*g* for 20 min at 4 °C. The resulting RNA pellet was washed once with 750 mL/L ethanol and dried for 10 min at room temperature. The pellet was dissolved in 25 μL of RNase-free water and stored at −80 °C until further processed. For RNA isolation from cells, total RNA was extracted from the cultured cells using Trizol reagent (Invitrogen, MA, USA) according to the manufacturer’s instructions.

**Table 1:** Demographic and clinical features of OLP patients and healthy controls in the present study

Variable	Training set			Validation set			HCMV titers set			Exosome set
	Cases	Controls	P value	Cases	Controls	P value	Cases	Controls	P value	Cases
Number	21	18		41	33		13	12		20
Age, years^a^	46.48 (9.08)	44.78 (4.18)	0.47^b^	46.59 (13.19)	46.45 (4.97)	0.96^b^	49.3 (6.2)	46.9 (5.5)	0.68^b^	45.6 (6.3)
Sex, n			0.75^c^			0.84^c^			0.87^c^	
Male	6 (29%)	6 (33%)		13 (32%)	12 (39%)		5 (38%)	5 (41%)		7 (35%)
Female	15 (71%)	12 (67%)		28 (68%)	21 (61%)		8 (62%)	7 (59%)		13 (65%)
Type of OLP, n										
Reticular OLP	10 (48%)			21 (51%)			7 (54%)			10 (50%)
Erosive OLP	11 (52%)			20 (49%)			6 (46%)			10 (50%)
Anti-HCMV IgG, n			0.27^c^			0.11^c^			0.29^c^	
Positive	21 (100%)	17 (94%)		41 (100%)	31 (94%)		13 (100%)	11 (92%)		20 (100%)
Negative	0 (0%)	1 (6%)		0 (0%)	2 (6%)		0 (0%)	1 (8%)		0 (0%)
Anti-HCMV IgM, n										
Positive	0 (0%)	0 (0%)		0 (0%)	0 (0%)		0 (0%)	0 (0%)		0 (0%)
Negative	21 (100%)	18 (100%)		41 (100%)	33 (100%)		13 (100%)	12 (100%)		20 (100%)
HCV, n			0.18^c^			0.04^c^			0.16^c^	
Positive	2 (9.5%)	0 (0%)		5 (12.2%)	0 (0%)		2 (15.4%)	0 (0%)		3 (15%)
Negative	19 (90.5%)	18 (100%)		36 (87.8%)	33 (100%)		11 (74.6%)	12 (100%)		17 (85%)
EBV, n						0.2^c^				
Positive	0 (0%)	0 (0%)		2 (4.9%)	0 (0%)		0 (0%)	0 (0%)		1 (5%)
Negative	21 (100%)	18 (100%)		39 (95.1%)	33 (100%)		13 (100%)	12 (100%)		19 (95%)
HIV, n										
Positive	0 (0%)	0 (0%)		0 (0%)	0 (0%)		0 (0%)	0 (0%)		0 (0%)
Negative	21 (100%)	18 (100%)		41 (100%)	33 (100%)		13 (100%)	12 (100%)		20 (100%)

^a^Age data are presented as the mean ± SD

^b^Student *t* test

^c^Two-sided χ^2^ test

### RT-qPCR analysis

We performed a TaqMan probe-based RT-qPCR assay according to the manufacturer’s instructions (Lightcycler^®^ 480 II, Roche) to investigate the differential expression of HCMV-encoded miRNAs between OLP patients and normal controls as described previously [24]. U6 small noncoding RNA (Applied Biosystems, Foster City, CA, USA) was used as a housekeeping gene to normalize the miRNA expression, and U6 expression levels were not significantly different between the two groups (Additional file 1: Figure S1). All reactions, including non-template controls, were performed in triplicate. The RT stem-loop primers and PCR primers (Applied Biosystems, Foster City, CA, USA) were highly specific for each target miRNA.

We assessed the detection limits of the RT-qPCR assay by conducting calibration curves developed with corresponding synthetic miRNA oligonucleotides (Additional file 1: Figure S2). All reactions were performed in triplicate. MiRNA expression in cells was also normalized to U6 small noncoding RNA. For mRNA quantification of ULBP1, total RNA was reverse transcribed into cDNA using oligo (dT). Real-time qPCR was performed using SYBR Green and normalized to GAPDH. The following PCR primers were used: ULBP1 forward: 5′-GTACTGGGAACAAATGCTGGAT-3′; ULBP1 reverse: 5′-AACTCTCCTCATCTGCCAGCT-3′; GAPDH forward, 5′-TGAAGCAGGCATCTGAGGG-3′; and GAPDH reverse, 5′-CGAAG GTGAAGAGTGGGAG-3′. The relative content of miRNA and mRNA were calculated using the 2^−∆Cq^ method and 2^−∆∆Cq^ method, respectively.

### HCMV titers

We tested the copy numbers of HCMV in peripheral blood leukocytes (PBLs) by quantitative PCR in an additional 13 OLP patients and 12 healthy control subjects. DNA from PBLs (separated by Haoyang, Tianjin, China) was extracted according to the manufacturer’s protocols of QIAamp DNA Mini kit (Qiagen, Hilden, Germany). We measured HCMV DNA using a TaqMan real-time PCR assay with the following HCMV-specific primers: HCMV DNA forward: 5′-CACGGTCCCGGTTTAGCA-3′, HCMV DNA reverse: 5′-CGTAACGTGGACCTGACGTTT-3′, FAM-labelled probe: 5′-FAM-TATCTGCCCGAGGATCGCGGTTACA-TAMRA-3′. Ten-fold diluted recombinant plasmid that contains the HCMV target sequence was used as a template for standard curve preparation (Additional file 1: Figure S3). Cq values were converted to absolute values from the standard curve. The 2-step thermocycling procedure consisted of 45 cycles of denaturation at 95 °C for 15 s, annealing and extension at 60 °C for 60 s. Results were expressed as copies per 1 mL blood.

### Determination of anti-HCMV IgG and IgM antibodies in plasma

Enzyme-linked immunosorbent assay (ELISA) was performed to detect anti-HCMV IgG and IgM antibodies in plasma using a HCMV IgG/IgM kit (MEDSON, NJ, USA) according to the manufacturer’s instructions. For the IgG-ELISA, a calibration curve, calibrated against the 1st WHO international standard, was used to quantitatively determine IgG antibody concentrations in each sample. For the IgM-ELISA, the test results were calculated using the optical density (OD) value at 450 nm, and the cut-off value for positivity was OD >1.2.

### Cell culture and luciferase reporter assays

The human embryonic kidney cell line HEK293 was obtained from the Shanghai Institutes for Biological Sciences (Shanghai, China). The complete growth medium of HEK293 was Dulbecco’s modified Eagle’s medium (DMEM) (Gibco, NY, USA) and 10% foetal bovine serum (Gibco, NY, USA). These cells were maintained at 37 °C under an atmosphere of 5% CO_2_—95% air. Luciferase reporter assays were performed to confirm that hcmv-miR-UL59 directly targeted the 3′UTR of the ULBP1 gene. The 3′UTR fragments from ULBP1 containing the predicted hcmv-miR-UL59 binding site were cloned into a pMIR-reporter plasmid (Ambion, Shanghai, China), and which was co-transfected into HEK293 cells with hcmv-miR-UL59 mimics by Lipofectamine 2000 (Thermo fisher, NY, USA). A β-galactosidase vector was co-transfected into HEK293 cells simultaneously as a transfection control. Cells were assayed using luciferase assay kits (Promega, Madison, WI, USA) at 24 h after transfection. The reported data represent three independent experiments.

### Western blot analysis

Total cell lysates (50 μg) from HEK293 cells were separated on 12% SDS-PAGE and transferred to polyvinylidenedifluoride (Bio-Rad, Hercules, CA, USA). The detailed protocol was described as following: briefly, after blocking in 20 mmol/L Tris–HCl, pH 7.6, containing 150 mmol/L NaCl, 0.1% Tween 20, and 5% (wt/vol) nonfat dry milk, the blots were incubated with the specific rabbit anti-human ULBP1 (Santa Cruz Biotechnology, CA, USA) and anti-GAPDH (Santa Cruz Biotechnology, CA, USA) overnight at 4 °C. The blots were then incubated with horseradish peroxidase conjugated secondary antibody (Santa Cruz Biotechnology, CA, USA) for 1 h while shaking at room temperature. The autoradiographic intensity of each band was scanned and quantified using Image J software.

### Exosome RNA extraction and quantification

Exosomes from the plasma samples of 20 OLP patients was isolated using the Total Exosome Isolation Kit (Invitrogen, MA, USA) according to the manufacturer’s instructions. Exosomal RNA was extracted using a miRNeasy Mini Kit (Qiagen, Hilden, Germany) according to the manufacturer’s instructions. Hcmv-miR-UL59 from exosomal RNA and plasma total RNA was quantified using RT-qPCR analysis. Ten-fold serial dilution of synthetic single-strand hcmv-miR-UL59 was used as a template for standard curve preparation (Additional file 1: Figure S2B). Cq values were converted to absolute values from the standard curve.

### Statistical analysis

Statistical analyses were performed using SPSS software (version 19.0). Data are presented as the mean ± SEM for miRNAs or mean ± SD for other variables. A 2-sided Student’s *t* test was used to compare differences in variables between groups. ANOVA analysis was performed when compared the plasma levels of the 5 upregulated miRNAs in different types of OLP patients. Univariate and multivariate logistic regression analyses were performed to analyse the associations between plasma HCMV encoded miRNAs and OLP. A *P* value of <0.05 was considered statistically significant.

## Results

### Expression profiles of HCMV-encoded miRNAs using RT-qPCR analysis

**Fig. 1:**
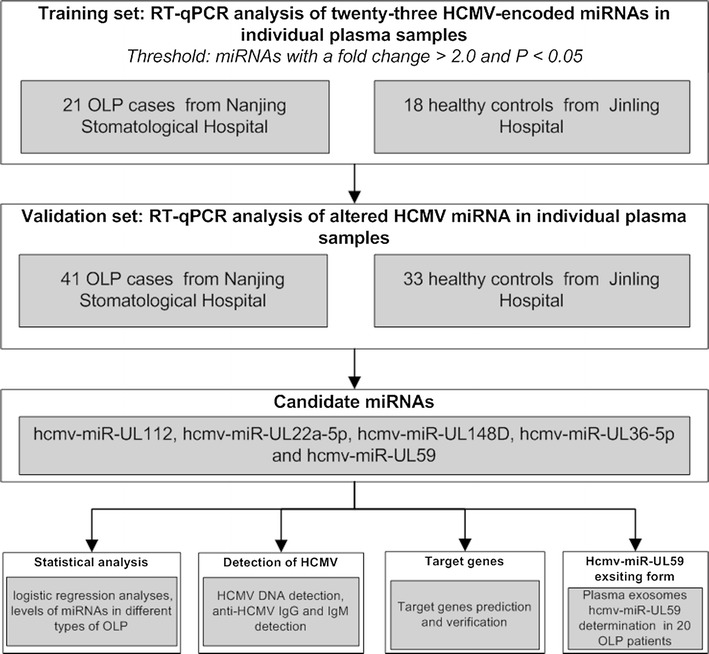
Overview of the experimental design

In the training set, 23 HCMV-encoded miRNAs were measured using a RT-qPCR assay in a cohort of individual plasma samples from 21 OLP patients and 18 healthy controls (Fig. 1). The RT-qPCR results showed that all of the examined 23 HCMV-encoded miRNAs were detectable in OLP patients and controls. Five of the 23 HCMV-encoded miRNAs, hcmv-miR-UL112-3p, hcmv-miR-UL22a-5p, hcmv-miR-UL148d, hcmv-miR-UL36-5p and hcmv-miR-UL59, were significantly upregulated in OLP samples compared with normal samples (fold change >2, P < 0.05) (Table 2). Our data suggest that the plasma expression pattern of HCMV-encoded miRNAs in OLP patients is different from healthy controls.

**Table 2:** Expression profile of HCMV-encoded miRNAs in OLP patients and normal controls in the training set

HCMV encoded miRNAs	OLP patients (n = 21)	Normal controls (n = 18)	Fold change	*P* value^a^
	Mean ± SEM	Mean ± SEM		
hcmv-miR-UL112-3p	3.76 ± 0.50	0.52 ± 0.06	7.20	2.711 × 10^−7^
hcmv-miR-UL112-5p	0.29 ± 0.03	0.15 ± 0.02	1.89	2.532 × 10^−3^
hcmv-miR-UL22a-3p	0.16 ± 0.02	0.13 ± 0.02	1.23	0.360
hcmv-miR-UL22a-5p	1.00 ± 0.23	0.37 ± 0.08	2.67	0.026
hcmv-miR-UL148d	53.70 ± 11.73	19.94 ± 4.94	2.69	0.017
hcmv-miR-UL36-5p	9.21 ± 2.07	1.72 ± 0.42	5.36	0.002
hcmv-miR-UL36-3p	0.33 ± 0.06	0.22 ± 0.04	1.50	0.131
hcmv-miR-UL59	3.98 ± 0.61	1.89 ± 0.25	2.11	2.034 × 10^−4^
hcmv-miR-UL69	0.02 ± 0.20 × 10^−2^	0.03 ± 0.01	0.76	0.308
hcmv-miR-UL70-5	1.29 ± 0.12	1.54 ± 0.17	0.84	0.244
hcmv-miR-US22-3p	0.53 ± 0.06	0.41 ± 0.07	1.28	0.192
hcmv-miR-US22-5p	8.01 ± 1.35	6.07 ± 0.72	1.32	0.225
hcmv-miR-US25-1	0.07 ± 0.01	0.05 ± 0.01	1.52	0.074
hcmv-miR-US25-2-3p	71.94 ± 67.34	2.57 ± 0.40	28.01	0.369
hcmv-miR-US25-2-5p	1.83 ± 0.27	1.23 ± 0.12	1.49	0.051
hcmv-miR-US29-3p	0.05 ± 0.01	0.08 ± 0.01	0.63	0.055
hcmv-miR-US33-3p	0.23 ± 0.02	0.20 ± 0.02	1.17	0.307
hcmv-miR-US33-5p	0.72 ± 0.08	0.78 ± 0.29	0.92	0.800
hcmv-miR-US4-3p	54.00 ± 46.89	1.11 ± 0.20	48.53	0.304
hcmv-miR-US4-5p	0.21 ± 0.03	0.31 ± 0.04	0.68	0.111
hcmv-miR-US5-1	2.03 ± 0.30	1.77 ± 0.40	1.15	0.495
hcmv-miR-US5-2-3p	0.04 ± 0.01	0.06 ± 0.01	0.72	0.040
hcmv-miR-US5-2-5p	3.60 ± 0.56	3.56 ± 0.38	1.01	0.800

Data are presented as the mean ± SEM

^a^Student *t* test

### Further confirmation of miRNAs

**Fig. 2:**
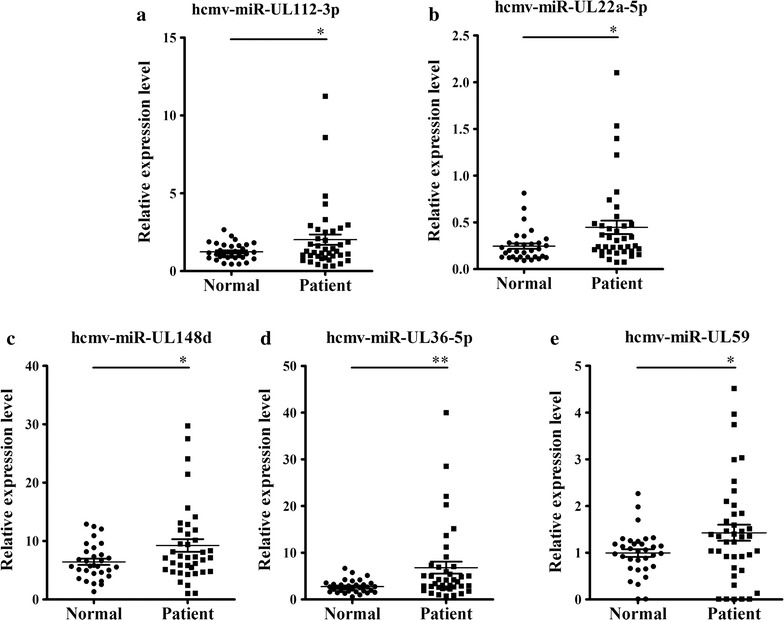
Plasma levels of 5 upregulated HCMV-encoded miRNAs in OLP patients in validation set. **a**–**e** In validation set, the relative expression levels of 5 HCMV-encoded miRNAs were measured in 40 OLP patients and 33 healthy controls using a RT-qPCR assay. The relative expression level of miRNAs was normalized to U6 expression. The relative content of miRNA was calculated using the 2^−∆Cq^ method. Each *P* value was derived from a 2-sided Student’s *t* test. *P < 0.05; **P < 0.01

**Fig. 3:**
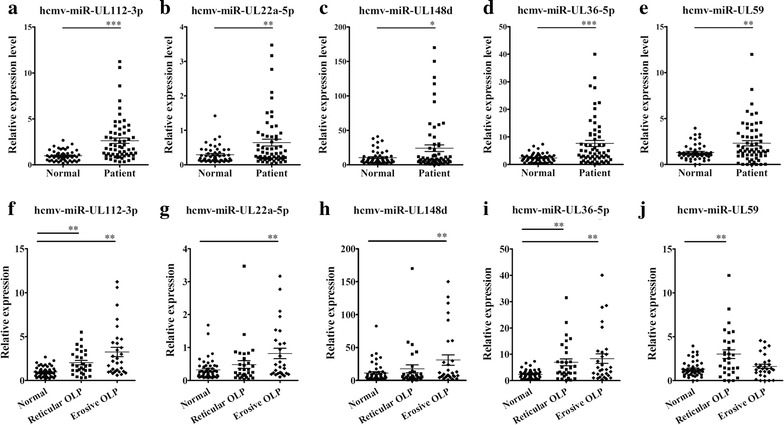
Plasma levels of the 5 upregulated miRNAs in different types of OLP. **a**–**e** In training set and validation set, the relative expression levels of 5 HCMV-encoded miRNAs were measured in 61 OLP patients and 51 healthy controls using a RT-qPCR assay, each P-value was derived from a 2-sided Student’s *t* test; **f**–**j** The relative expression levels of 5 upregulated HCMV-encoded miRNAs in 30 erosive OLP patients and 31 reticular OLP patients and 51 healthy controls were measured using RT-qPCR, each P value was derived from ANOVA analysis. The relative content of miRNA was calculated using the 2^−∆Cq^ method. The relative expression of miRNAs was normalized to U6 expression. *P < 0.05; **P < 0.01

Subsequently, the 5 selected miRNAs were further confirmed in an additional larger cohort of 40 OLP patients and 33 healthy controls (Fig. 1). Consequently, the alterations of the expression patterns of these 5 miRNAs in the OLP samples in this validation cohort were consistent with the results from the former cohort (Fig. 2; Additional file 1: Table S1). The differences in relative expression levels for the 5 altered miRNAs in all 61 OLP patients and 51 control individuals in the two cohorts are shown in Fig. 3a–e and Additional file 1: Table S2.

### Plasma levels of the 5 identified miRNAs in different types of OLP

Oral lichen planus is categorized into several clinical forms. The most common categories of OLP are reticular OLP and erosive OLP. These two types of OLP are obviously distinguished from each other in clinical features, and the development of these diseases are also discrepant [25]. We evaluated changes in the plasma levels of the aforementioned 5 miRNAs in OLP patients with different clinical types who enrolled in the training and validation cohorts (n = 61). As showed in Fig. 3f–j, hcmv-miR-UL112-3p and hcmv-miR-UL36-5p were upregulated in both reticular OLP and erosive OLP, whereas hcmv-miR-UL22a-5p and hcmv-miR-UL148d were only significantly upregulated in erosive OLP when compared with the normal controls. In addition, hcmv-miR-UL59 were only elevated in reticular OLP compared with the normal controls (Fig. 3j). However, no obvious difference in the levels of the 5 miRNAs was observed between the two different types of OLP (Fig. 3f–j; Additional file 1: Table S3).

### Elevated levels of HCMV encoded miRNAs in plasma are associated with the presence of OLP

To evaluate the clinical usefulness of the five altered HCMV encoded miRNAs in plasma for OLP patients, we first performed a forward stepwise binary logistic regression using the status of OLP as the dependent variable. When using the control group as the reference category, three of the 5 selected miRNAs were independently correlated with OLP (Additional file 1: Table S4). The odds ratios (ORs) of these miRNAs for OLP were as follows: hcmv-miR-UL112-3p (OR = 6.044, 95% CI 1.659–22.024, P = 0.006), hcmv-miR-UL36-5p (OR = 5.565, 95% CI 1.520–20.374, P = 0.010), hcmv-miR-UL59 (OR = 5.565, 95% CI 1.520–20.374, P = 0.010), hcmv-miR-UL22a-5p (OR = 2.370, 95% CI 0.595–9.447, P = 0.747) and hcmv-miR-UL148d (OR = 3.077, 95% CI 0.799–11.852, P = 0.501). Furthermore, after adjusting for age, gender, CMV IgG, CMV IgM, HIV, HCV and EBV, hcmv-miR-UL112-3p still remained an independent association with OLP by multivariate logistic regression analyses using a dependent two-category variable (Additional file 1: Table S4).

### Detection of HCMV

**Fig. 4:**
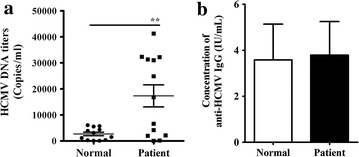
Detection of HCMV and its relationship with HCMV-encoded miRNAs. **a** The HCMV DNA titers were calculated in PBLs of OLP patients and compared with healthy controls; **b** Comparison of the concentrations of anti-HCMV IgG in the OLP group (n = 61) versus the control group (n = 51). *P < 0.05; **P < 0.01

Because the levels of the 5 identified HCMV-encoded miRNAs were significantly upregulated in plasma from OLP patients, we investigated whether the latent infection of HCMV in OLP patients was different from that in the healthy controls. It has been reported that PBL-derived HCMV DNA has the higher sensitivity in defining HCMV infection than plasma HCMV DNA in latency [26, 27]. Therefore, we detected HCMV DNA in PBLs of additional 13 OLP patients and 12 healthy controls using a quantitative PCR assay, and found that the HCMV DNA was significantly higher in OLP patients than in control subjects, with the concentrations were (56.01–41,316) ± 15,311 copies/mL in OLP and (77.43–6065) ± 2368 copies/mL in controls, respectively (Fig. 4a). Furthermore, we conducted ELISA analyses to measure the concentrations of anti-HCMV IgG and IgM in samples from both the training and validation sets. Our results revealed that almost all the subjects in both groups were anti-HCMV IgG positive and no significant differences were found in the plasma concentrations of anti-HCMV IgG between groups (Fig. 4b), neither the plasma positivity of anti-HCMV IgG and anti-HCMV IgM (100.00% versus 93.65%, *P* = 1.0; 0% versus 0%, *P* = 1.0, respectively). In addition, we also assessed the HCMV immediate early gene 1 (IE1) mRNA levels using RT-qPCR assay in the above patients and controls, nevertheless, none IE1 signal could be detected in both the blood of OLP patients and healthy controls.

### ULBP1 as a direct target of HCMV-miR-UL59

**Fig. 5:**
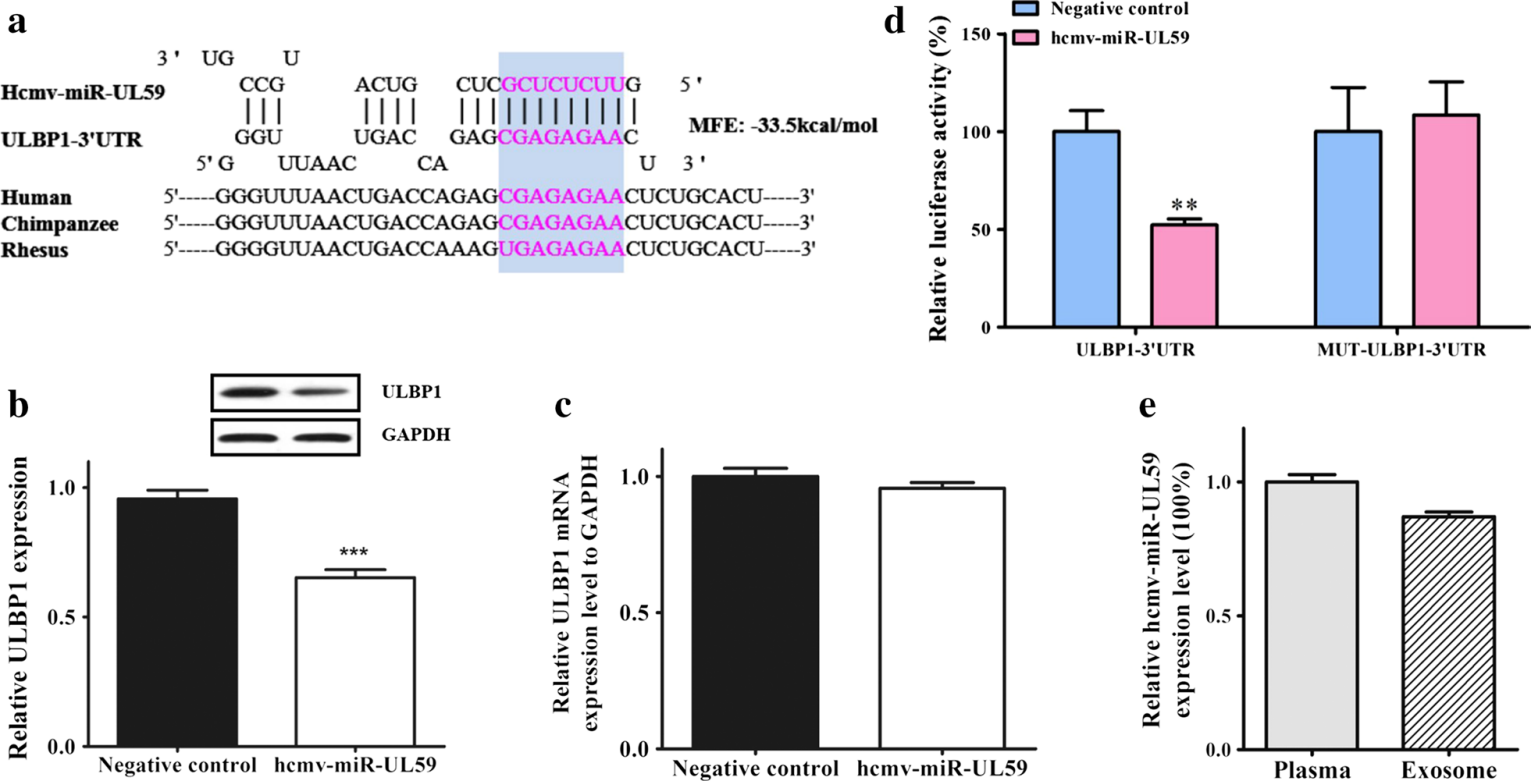
Direct target and the distribution of HCMV-miR-UL59 in exosomes. **a** The predicted binding site for hcmv-miR-UL59 in the 3′UTR of ULBP1 mRNA; **b** Western blotting analysis showed that the protein level of ULBP1 was downregulated in HEK293 cells transfected with hcmv-miR-UL59 mimics; **c** Real-time qPCR analysis showed ULBP1 mRNA level in HEK293 cells was not affected by hcmv-miR-UL59 overexpression; **d** Hcmv-miR-UL59 downregulated luciferase activity in HEK293 cells transfected with a pMIR-report plasmid that contained the 3′UTR of ULBP1 including HCMV-miR-UL59 binding site, whereas luciferase activity was not altered in cells transfected with MUT-ULBP1-3′UTR; **e** Absolute concentration of hcmv-miR-UL59 in plasma and exosome portions from 20 OLP patients using RT-qPCR analysis. *P < 0.05; **P < 0.01; ***P < 0.001

To delineate the molecular mechanism underlying the role of dysregulated HCMV-encoded miRNAs in OLP, we used three algorithms, miR-Tar, miRanda and RNA hybrid, to locate HCMV miRNAs seed matches within human genes and HCMV genes to identify potential mRNA targets of these upregulated HCMV-encoded miRNAs in OLP patients. Computational predictions indicated that some potential target genes of the 5 identified HCMV-encoded miRNAs are involved in immune regulation or viral replication or latency in HCMV or the regulation of apoptosis, tumours and cytokines in humans (Additional file 1: Table S5). Among these candidate targets, cytomegalovirus UL16-binding protein 1 (ULBP1), which is an NKG2D ligand, was predicted to be a potential target of hcmv-miR-UL59 (Additional file 1: Table S5). One predicted hybridization was observed between hcmv-miR-UL59 and the 3′-UTR of ULBP1, with perfect base-pairing between the seed region and the cognate target (Fig. 5a). The minimum free energy value of the hybridization between hcmv-miR-UL59 and ULBP1 is −33.5 kcal/mol, which is well within the range of genuine miRNA-target pairs (Fig. 5a). The hcmv-miR-UL59 binding sequence in the ULBP 3′-UTR was also highly conserved across primate species (Fig. 5a). The correlation between hcmv-miR-UL59 and ULBP1 was examined by evaluating ULBP1 expression in HEK293 cells after hcmv-miR-UL59 overexpression. The efficient overexpression of hcmv-miR-UL59 in HEK293 cells is shown in the Additional file 1: Figure S4. As expected, the transfection of HEK293 cells with hcmv-miR-UL59 mimics significantly decreased the ULBP1 protein levels (Fig. 5b), whereas the overexpression of hcmv-miR-UL59 did not affect ULBP1 mRNA stability (Fig. 5c). Furthermore, we performed a luciferase reporter assay by transfecting HEK293 cells with a reporter vector that consisted of a luciferase gene containing the wild-type or mutated sequence of the 3′UTR of ULBP1, including the hcmv-miR-UL59 binding site in its 3′UTR region, to confirm whether hcmv-miR-UL59 directly regulates ULBP1 (Fig. 5d). This experiment demonstrated that transfection with the WT-ULBP1-3′UTR plasmid inhibited approximately 50% of WT-ULBP1-3′UTR luciferase activity (100% versus 52.27%, P < 0.01), but transfection with MUT-ULBP1-3′UTR did not significantly alter luciferase activity (100% versus 108.45%, P > 0.05) (Fig. 5d). Taken together, these results confirm that ULBP1 is a direct and bona fide target of hcmv-miR-UL59.

### Plasma HCMV-miR-UL59 is primarily encapsulated in exosomes

We suspected that plasma hcmv-miR-UL59 is primarily encapsulated in membrane-bound vesicles. To test our hypothesis, we determined the extent of plasma hcmv-miR-UL59 association with exosomes that were isolated from 20 OLP plasma samples. We observed that approximately 86% of plasma hcmv-miR-UL59 was contained in the exosome pellet (Fig. 5e). Furthermore, in order to confirm that hcmv-miR-UL59 was really encapsulated in exosomes, we firstly digested the isolated exosomes with RNase H for 1 h and then determined the levels of hcmv-miR-UL59 using qRT-PCR analysis, and found that the content of HCMV-miR-UL59 was not decreased after RNase treatment when compared with controls. Next, to test whether the exosomes protect the hcmv-miR-UL59 from degradation by RNase H, we disrupted the exosomes by adding 0.1% Triton X-100 during the RNase H treatment. As shown in Additional file 1: Figure S5, the levels of hcmv-miR-UL59 were significantly decreased, which support our hypothesis that hcmv-miR-UL59 is associated with exosomes.

## Discussion

Human cytomegalovirus infects most people worldwide, and it establishes a lifelong latency that must be suppressed by the immune system [28]. Cytomegalovirus is a clinically important pathogen that is associated with high levels of morbidity and mortality in immunocompromised individuals [29]. The majority of HCMV infections are asymptomatic, but these infections challenge our immune system by directly or indirectly inhibiting the function of some immune cells, particularly NK cells, and dysregulating cellular chemokines to their benefit [14, 30]. HCMV may also be involved in the pathogenesis of diseases of unknown origin. In particular, Li et al. identified the association between HCMV infection and the development of essential hypertension through the dysregulated expression of hcmv-miR-UL112-3p [18]. Beside the above report, higher prevalence of hcmv-miR-UL112-3p was also detected in patients with diabetes mellitus and glioblastoma multiforme [19]. Pan et al. showed that serum HCMV-miR-US4-1 can serve as a novel biomarker for predicting the outcome of interferon α treatment in chronic hepatitis B patients [21]. Thus the availability of a highly sensitive test capable of detecting HCMV infection is of great significance. However, direct links between HCMV infection and OLP remain undefined. In this study, we examined the HCMV DNA in PBLs from our studied OLP patients, and the HCMV DNA in PBLs was significantly higher in OLP patients than in control subjects, while the positive rates of anti-HCMV IgG (past or current infection) and anti-HCMV IgM (current or recent infection) were not different between OLP patients and controls. Interestingly, HCMV-encoded miRNAs were detectable in plasma samples from both OLP patients and controls. Notably, the plasma profile of HCMV-encoded miRNAs in OLP patients was different from normal controls, and 5 of 23 examined HCMV-encoded miRNAs were significantly upregulated in OLP patients. These results suggest that OLP patients have high loads of latent HCMV genomes and high levels of specific HCMV miRNAs compared to HCMV seropositive controls. In addition, our subsequent bioinformatics combined with luciferase analyzes and western blotting assays demonstrated that one of the HCMV encoded miRNA, hcmv-miR-UL59, could target ULBP-1, for which is a critical protein in immune elimination of HCMV. Thus, we suspected that HCMV may be involved in the pathogenesis of OLP, and those individuals with high latent loads of HCMV are perhaps more prone to OLP, nevertheless, further study are needed.

Notably, the majority of the 5 miRNAs identified in our study have been reported to be related to some HCMV infection diseases, for instance, hcmv-miR-UL22a-5p may be a potential predictor of clinical and virological endpoint in solid organ transplant patients [31], and increased hcmv-miR-UL112-3p was also reported to be related with glioblastoma, rheumatoid arthritis, diabetes mellitus essential hypertension as well as congenital infection [18, 19, 32]. All these results indicate that these HCMV encoded miRNAs may share similar physiological and pathological roles in HCMV infection related diseases as well as in OLP, eventhough much further study is needed to confirm this. On account of the unclear pathogenesis of OLP, our results may provide a clue to research the aetiology of OLP and insight into the role of circulating HCMV encoded miRNAs in HCMV infection in vivo.

Human cytomegalovirus has evolved a variety of mechanisms to evade host immune surveillance, which provides this virus with the capability of lifelong persistence in infected hosts in the form of a latent infection with the ability to reactivate when immune surveillance is compromised [33]. This study demonstrated that hcmv-miR-UL59 directly targeted UL16-binding protein 1 (ULBP1), which is a NKG2D ligand [33]. NKG2D is a key NK activating receptor that recognizes a family of stress-induced ligands, including MICA, MICB, and ULBP1-6 [34]. ULBP1 is induced by cellular stress and presented on the cell surface when cells are infected with HCMV, and ULBP1 interacts with NKG2D to mediate the killing of virus-infected cells by NK cells [14, 35]. Simultaneously, the HCMV glycoprotein UL16 binds to intracellular ULBP1 and inhibits its expression at the cell surface, which reduces the susceptibility of virus-infected cells to cytotoxic destruction by NK cells [36–38]. Hcmv-miR-UL59 likely performs a congruent function as HCMV glycoprotein UL16. In addition, since ULBP1 is a critical protein in immune elimination of HCMV and also a novel potential target of HCMV-miR-UL-59, we speculated that intensive research on the functions of this newly regulatory relationship will help us to uncover the molecular mechanism of HCMV infection in future.

Exosomes are the major ‘miRNA transporter’ between cells for regional or long-distance communication [39, 40]. The vesicular structures contain membrane proteins similar to those of the donor cell and contain miRNA derived from their donor cell cytoplasm. They can be taken up and transfer their content to modulate cellular activities in recipient cells. Thus, the encapsulated miRNA have the ability to signal and regulate their target genes within the local microenvironment as well as at a distance [41]. The possible role of hcmv-miR-UL59 in the immunoevasion mechanism of HCMV suggests that hcmv-miR-UL59 is primarily entrapped in exosomes in plasma. We found that most hcmv-miR-UL59 (86%) that was detectable in plasma samples from OLP patients was packed in exosomes during HCMV latency. Therefore, we speculated that the presence of hcmv-miR-UL59 and other upregulated HCMV-encoded miRNAs in the plasma of OLP patients might be a result of secreted exosomes from infected or inflammatory cells in which the virus reside. These cells may not secrete the HCMV but may secrete the HCMV encoded miRNAs into the circulation due to ongoing virus replication inside the cells in HCMV latency. These circulating miRNAs may be of some indicative value in HCMV infection.

The advantages of a miRNA-based regulation mechanism are multiple. These molecules are small, simpler to develop than a regulatory protein [42]. Furthermore, miRNAs are non-immunogenic molecules that allow viruses to regulate the expression of their host genome and establish an optimal environment while remaining undetected [43]. Unfortunately, we did not illuminate the specific underlying pathophysiological mechanisms between HCMV-encoded miRNAs and OLP. Hcmv-miR-UL112-3p targets MICB and IRF-1, which play critical roles in immunological, inflammatory, and anti-infection responses, and these proteins have a close relationship with essential hypertension [18, 44]. Hcmv-miR-UL148d downregulates IEX-1 expression, which contributes to the anti-apoptotic effects caused by ectopically expressed IEX-1 [45]. The chemokine RANTES (regulated on activation, expressed and secreted by normal T-cell) is a direct target of hcmv-miR-UL148d, which provides a potential mean for immunosuppressive therapy against HCMV [29]. Despite these findings, the targets of HCMV-encoded miRNAs were not fully identified, and much data about their functions during infection and their relationship with human diseases are yet to be known.

## Conclusions

In summary, we found that the expression profile of HCMV-encoded miRNAs in plasma samples from patients with OLP was different from that in normal controls, and 5 HCMV-encoded miRNAs were highly upregulated in OLP patients. Furthermore, we discovered that hcmv-miR-UL59 could target the cellular gene ULBP1, which is capable of mediating the immune elimination of HCMV-infected cells. A full understanding of the distinct express pattern of HCMV-encoded miRNAs and its relationship with OLP in vivo may lead to a broad clinical application.

## Additional file

**Additional file 1.** **Additional method.** Quantification of miRNAs by TaqMan probe-based RT-qPCR. **Figure S1.** Cq Values of U6 in plasma samples from 61 OLP patients and 51 healthy controls. **Figure S2.** Standard curves of 5 HCMV encoded miRNAs. **Figure S3.** Standard curve of recombinant plasmid that contained the HCMV target sequence. **Figure S4.** The overexpression efficiency of hcmv-miR-UL59 in HEK293 cells. **Figure S5.** The relative expression levels of hcmv-miR-UL59 in plasma exosome, RNase H treated exosome and Triton/RNase H treated exosome. **Table S1.** The relative expression levels of HCMV-encoded miRNAs in OLP patients and normal controls in the validation set. **Table S2.** The relative expression levels of HCMV-encoded miRNAs in OLP patients and normal controls. **Table S3.** The relative expression levels of HCMV-encoded miRNAs in the two different types of OLP patients and normal controls. **Table S4.** Univariate and multivariate logistic regression analyses of plasma HCMV miRNAs for OLP. **Table S5.** Targets of HCMV-encoded miRNAs.

## Abbreviations

OLP: oral lichen planus
miRNA: microRNA
HCMV: human cytomegalovirus
RT-qPCR: quantitative reverse transcription polymerase chain reaction
Cq: quantification cycle
ELISA: enzyme linked immunosorbent assay
