# Supplementary material for: Distinct expression profile of HCMV encoded miRNAs in plasma from oral lichen planus patients
Source: J Transl Med. 2017 Jun 7;15:133. doi: 10.1186/s12967-017-1222-8 (PMC5463403; doi:10.1186/s12967-017-1222-8)
Supplement: Supplementary file 1 — Additional file 1. Additional method. Quantification of miRNAs by TaqMan probe-based RT-qPCR. Figure S1. Cq Values of U6 in plasma samples from 61 OLP patients and 51 healthy controls. Figure S2. Standard curves of 5 HCMV encoded miRNAs. Figure S3. Standard curve of recombinant plasmid that contained the HCMV target sequence. Figure S4. The overexpression efficiency of hcmv-miR-UL59 in HEK293 cells. Figure S5. The relative expression levels of hcmv-miR-UL59 in plasma exosome, RNase H treated exosome and Triton/RNase H treated exosome. Table S1. The relative expression levels of HCMV-encoded miRNAs in OLP patients and normal controls in the validation set. Table S2. The relative expression levels of HCMV-encoded miRNAs in OLP patients and normal controls. Table S3. The relative expression levels of HCMV-encoded miRNAs in the two different types of OLP patients and normal controls. Table S4. Univariate and multivariate logistic regression analyses of plasma HCMV miRNAs for OLP. Table S5. Targets of HCMV-encoded miRNAs. [file 12967_2017_1222_MOESM1_ESM.doc]

**Additional files**

**The distinct expression profile of HCMV encoded miRNAs in plasma from oral lichen planus patients**

¶First Author: Meng Ding, Xiang Wang, Cheng Wang

*** Correspondence:**

Chunni Zhang: [zchunni27@hotmail.com](mailto:zchunni27@hotmail.com);

Ke Zen: [kzen@nju.edu.cn](mailto:kzen@nju.edu.cn).

Wenmei Wang: wenmei-wang @hotmail.com;

**Additional methods**

**Quantification of miRNAs by hydrolysis-based RT-qPCR**

Hydrolysis probe-based RT-qPCR assay of serum or seminal plasma was performed according to the manufacturer’s instructions (Applied Biosystems) with a minor modification. Briefly, the reverse transcription reaction was carried out in 10 μL containing 2 μL of extract RNA, 1 μL of 10 mmol/L dNTPs, 0.5 μL of AMV reverse transcriptase (TaKaRa) , 1 μL of a stem-loop RT primer (Applied Biosystems), 2 μL of 5×reverse transcription buffer and 3.5 μL of diethylpyrocarbonate (DEPC) water. For synthesis of cDNA, the reaction mixtures were incubated at 16^o^C for 30 min, at 42^o^C for 30 min, at 85^o^Cfor 5 min, and then held at 4^o^C.Real-time PCR was performed (1 cycle of 95^o^C for 5 min, and 40 cycles of 95^o^C for 15 sec and 60^o^C for 1 min) with anLightcycler® 480 IIDetection System. The reaction was performed with a final volume of 20 μL containing 1 μL of cDNA, 0.3 μL of Taq, 0.33 μL of hydrolysis probe (Applied Biosystems), 1.2 μL of 25 mmol/L MgCl_2_, 0.4 μL of 10 mmol/L dNTPs, 2 μL of 10×PCR buffer, and 14.77 μL of DEPC water. All reactions, including no-template controls, were performed in triplicate. The resulting Cq values were determined using fixed threshold settings and U6 small noncoding RNA (Applied Biosystems, Foster City, CA, USA) was used as a housekeeping gene to normalize the miRNA expression.

For each assay, calibration curves were prepared by ten-fold serial dilution of synthetic single-strand miRNAs for the five HCMV encoded miRNAs (including HCMV-miR-UL59, HCMV-miR-UL148d, HCMV-miR-UL22a-5p, HCMV-miR- UL112 and HCMV-miR-UL36-5p) synthesized by TaKaRa, Dalian, China from 10 fM/L to 10^5^pM/L, and the levels of the synthetic miRNAs were assessed by RT-qPCR assay. The resulting Cq values were plotted versus the log_10_ of the amount of the synthetic miRNAs. Each sample and each dilution of the calibrators were run in triplicate for analysis. All data were collected and analyzed with aLightcycler® 480 II Detection System (Roche, Basel, Swit). Every batch of amplifications included three water blanks as negative controls for each of the reverse transcription and the PCR steps. Employing the standard curves to calculate the absolute concentrations of five HCMV encoded miRNAs. The limit of detection and dynamic range for each miRNA was 10^2^ fmol/L and 10^2^ fmol/L～1×10^5^ pmol/L (hcmv-miR-UL59), 10 fmol/L and 10 fmol/L～1×10^5^ pmol/L (hcmv-miR-UL148d), 10^2^ fmol/L and 10^2^ fmol/L～1×10^5^ pmol/L (hcmv-miR-UL22a-5p), 10^2^ fmol/L and 10^2^ fmol/L～1×10^5^ pmol/L (hcmv-miR-UL112), 10^3^ fmol/L and 10^3^ fmol/L～1×10^5^ pmol/L (hcmv-miR-UL36-5p).

**Additional data**


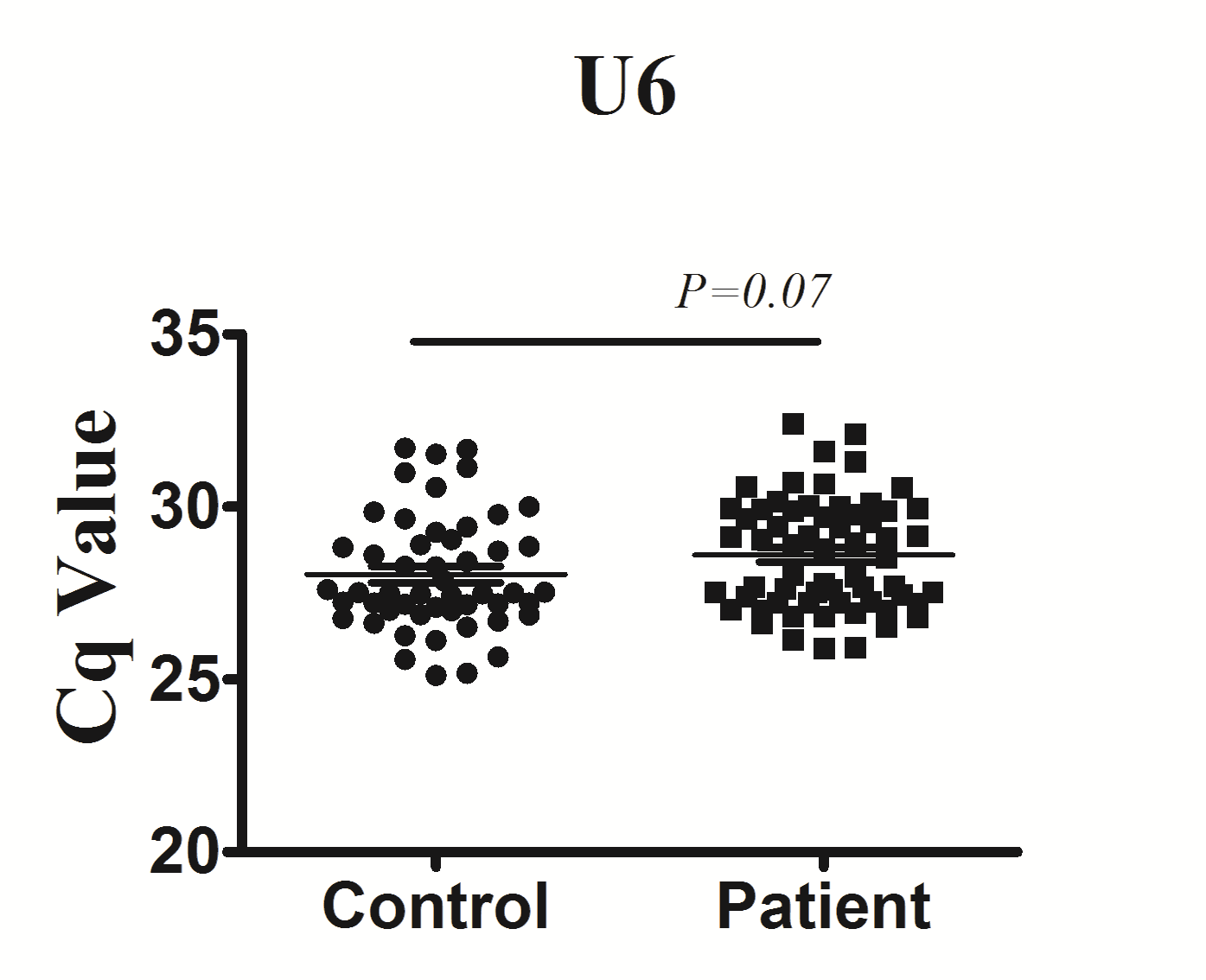


**Figure S1.** **Cq Values of U6 in plasma samples from 62 OLP patients and 51 healthy controls.**


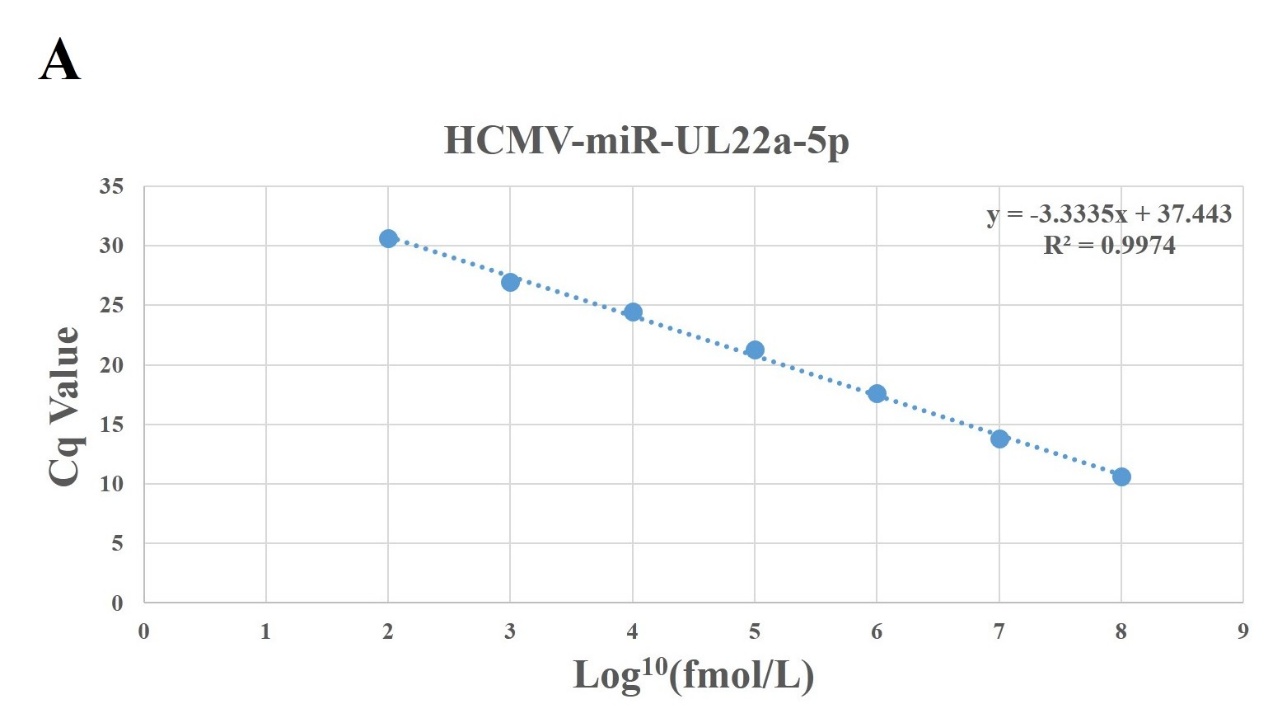


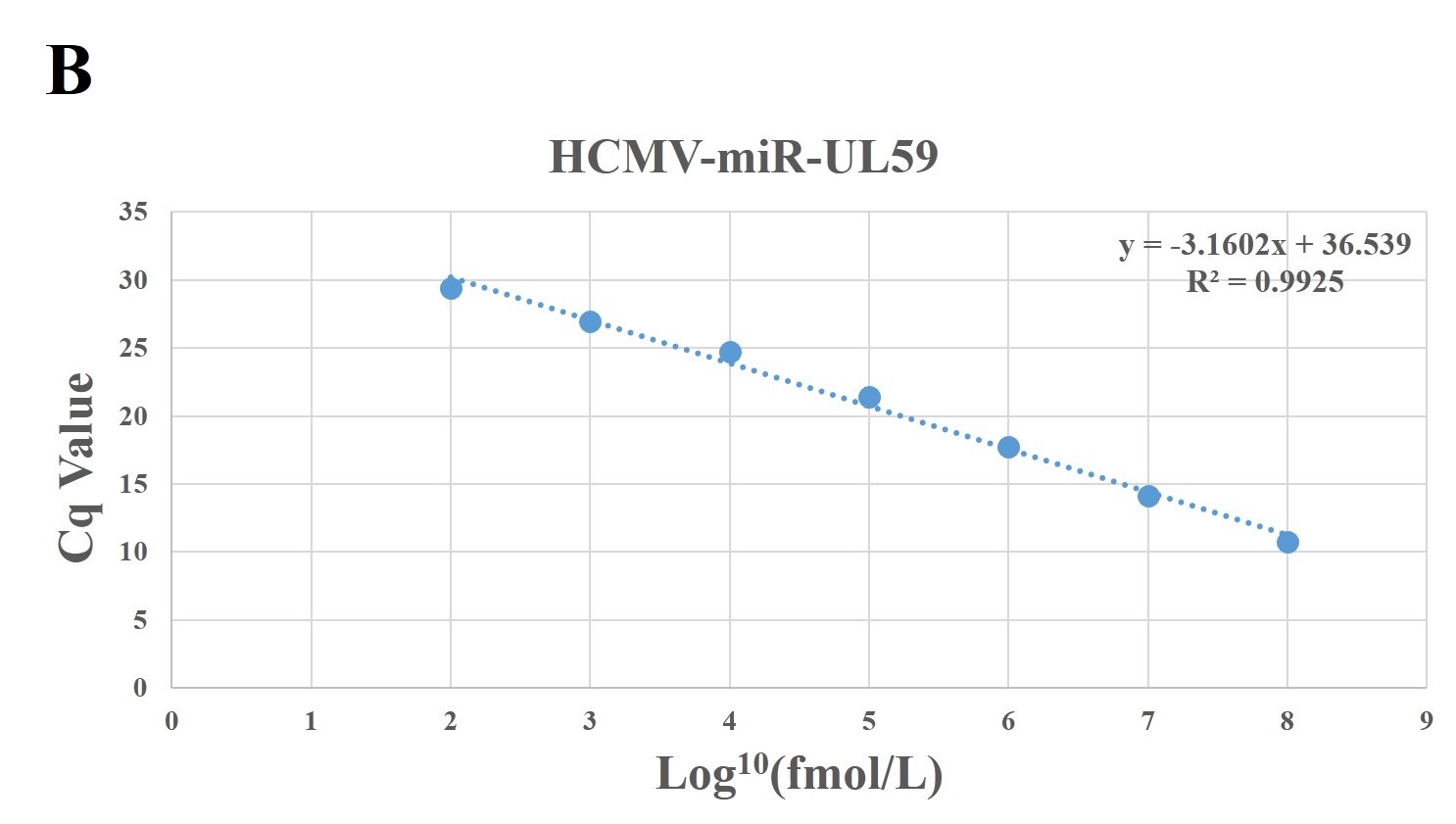


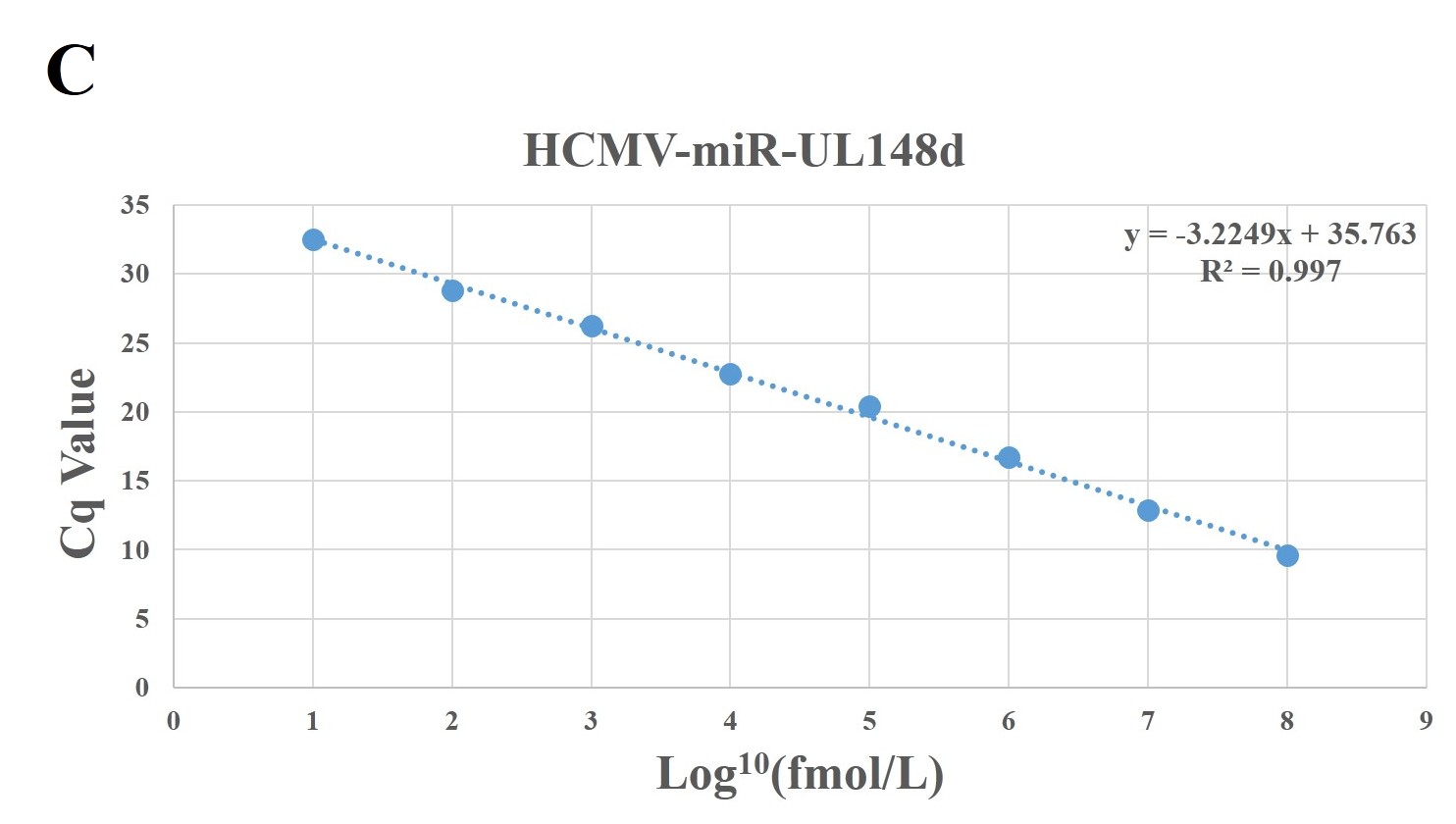


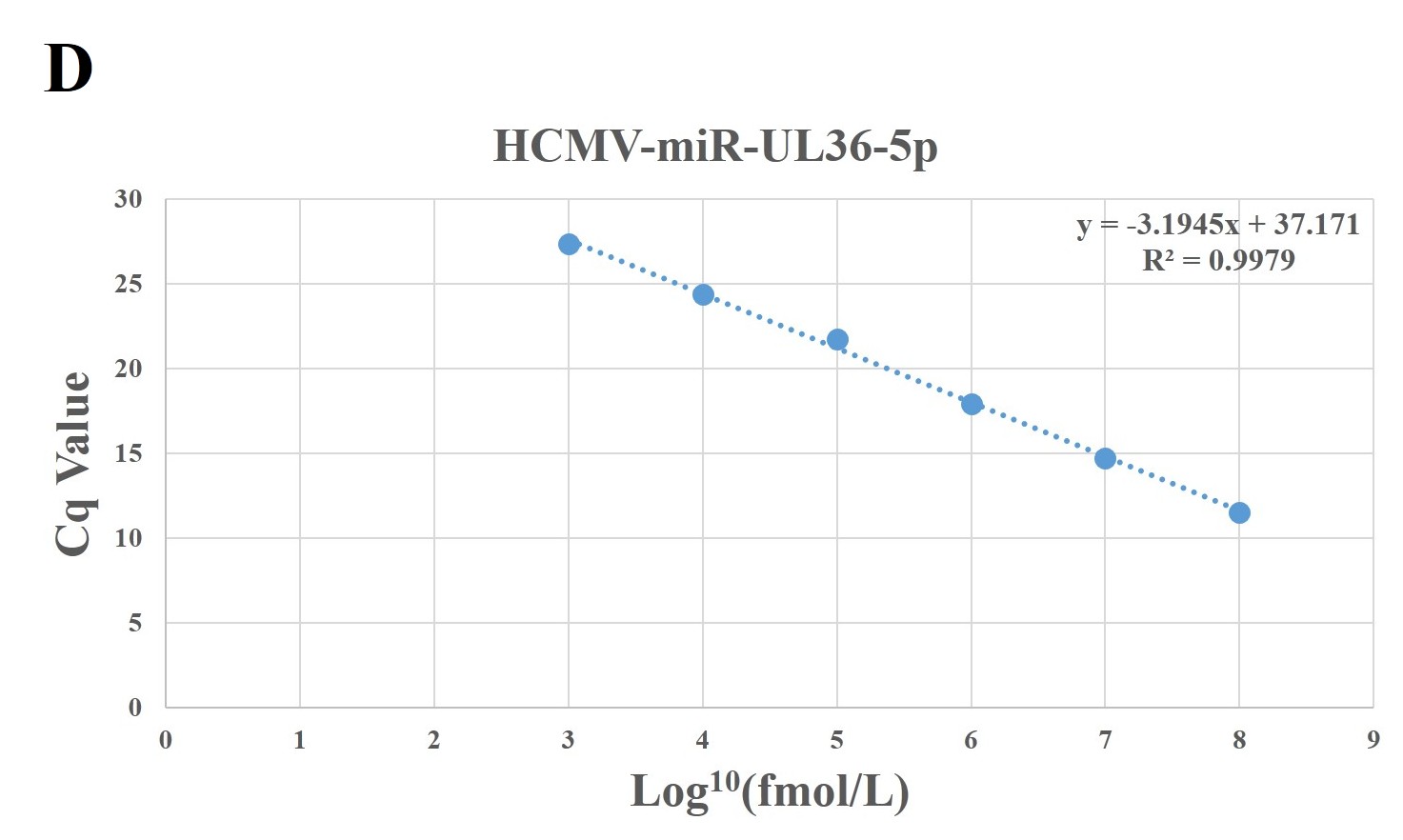


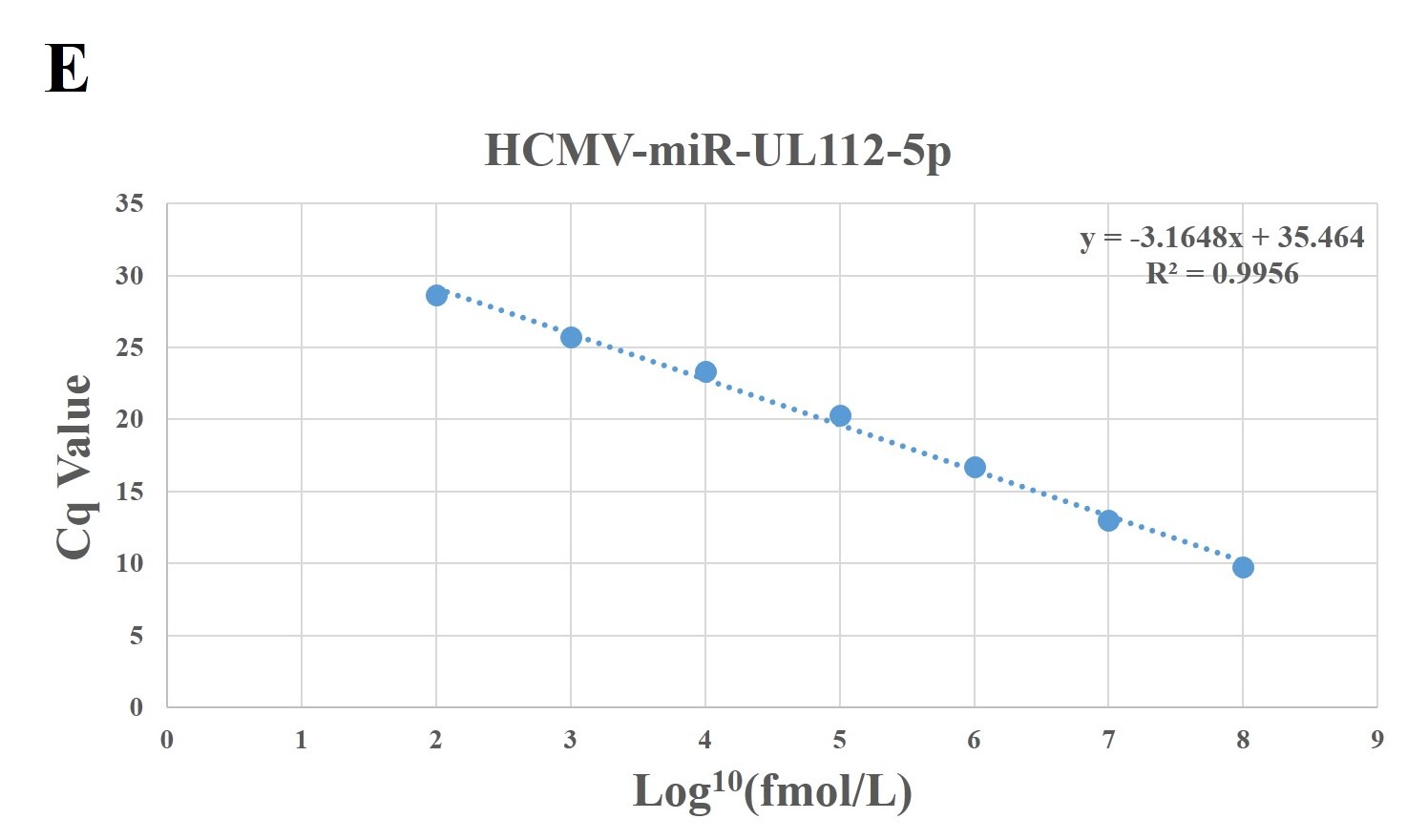


**Figure S2.** **Standard curves of 5 HCMV encoded miRNAs.** (A - E) Standard curves of HCMV-miR-UL22a-5p, HCMV-miR-UL59, HCMV-miR-UL148d, HCMV-miR-UL36-5p and HCMV-miR-UL112-5p using synthetic miRNAs.


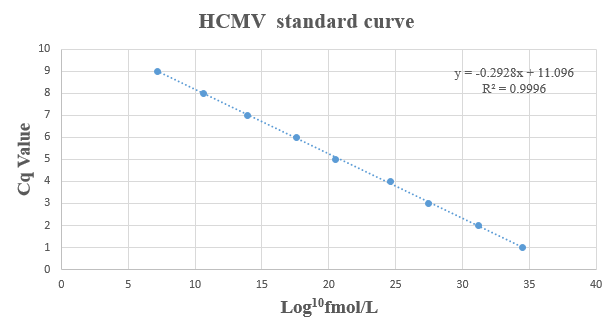


**Figure S3. Standard curve of recombinant plasmid that contained the HCMV target sequence.**


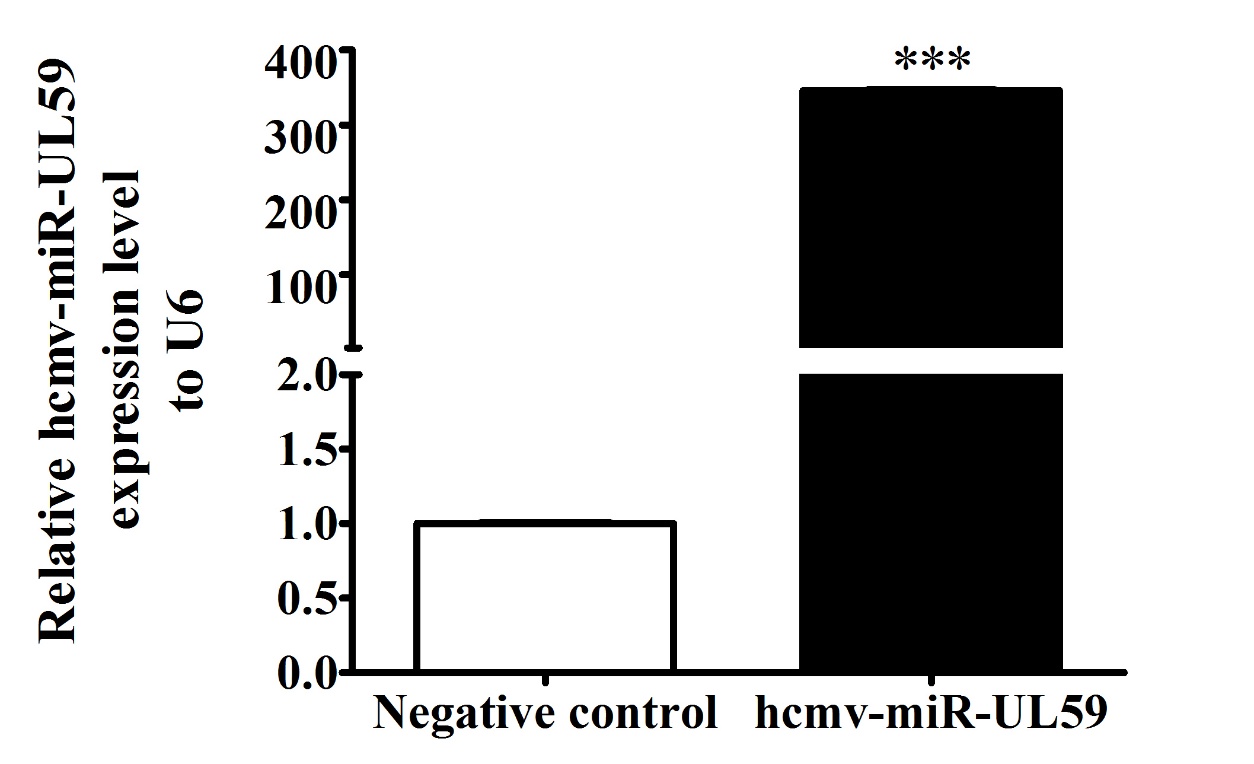


**Figure S4. The overexpression efficiency of hcmv-miR-UL59 in HEK293 cells.** The expression levels of mature hcmv-miR-UL59 in HEK293 cells transfected with hcmv-miR-UL59 mimics. ****P* < 0.001.


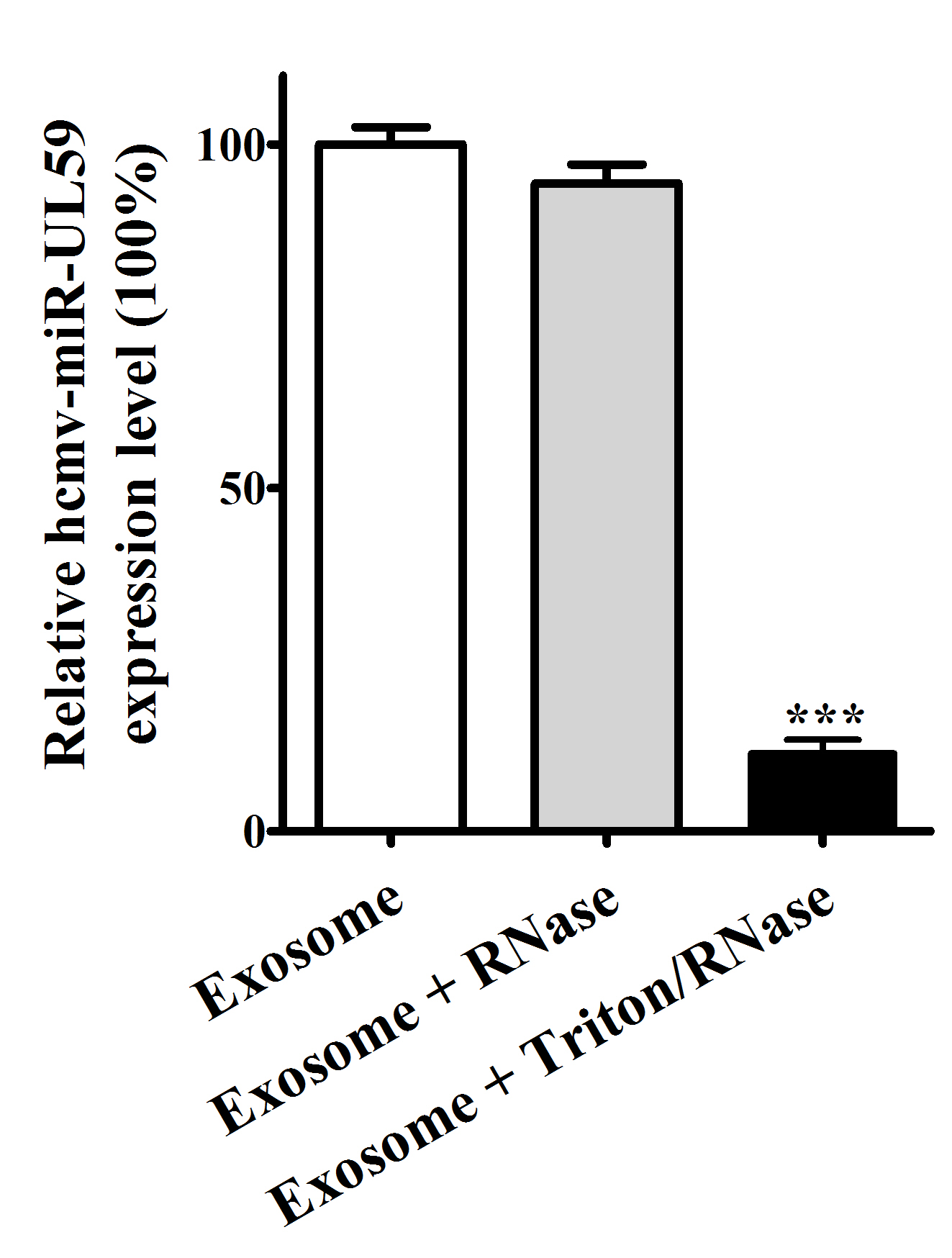


**Figure S5. The relative expression level of hcmv-miR-UL59 in plasma exosome.** The exosomes isolated from plasma of OLP patients were treated with RNase H or Triton/ RNase H. ****P* < 0.001.

**Table S1** Relative expression level of HCMV-encoded miRNAs in OLP patients and normal controls in the validation set^a^

| **HCMV encoded miRNAs** | **OLP patients(n=40)** | **Normal controls(n=33)** | **fold change** | ***P* value^b^** |
| --- | --- | --- | --- | --- |
|  | **Mean ± SEM** | **Mean ± SEM** |  |  |
| hcmv-miR-UL112-3p | 2.02 ± 0.34 | 1.23 ± 0.09 | 1.64 | 0.040 |
| hcmv-miR-UL22a-5p | 0.45 ± 0.07 | 0.24 ± 0.03 | 1.87 | 0.017 |
| hcmv-miR-UL148d | 9.24 ± 1.07 | 5.44 ± 0.53 | 1.69 | 0.033 |
| hcmv-miR-UL36-5p | 6.80 ± 1.29 | 2.75 ± 0.24 | 2.45 | 0.006 |
| hcmv-miR-UL59 | 1.63 ± 0.17 | 0.99 ± 0.08 | 1.64 | 0.032 |

^a^ Data are presented as the Mean ± SEM

^b^ Student-t test.

**Table S2** Relative expression level of HCMV-encoded miRNAs in OLP patients and normal controls^a^

| **HCMV encoded miRNAs** | **OLP patients(n=61)** | **Normal controls(n=51)** | **fold change** | ***P* value^b^** |
| --- | --- | --- | --- | --- |
|  | **Mean ± SEM** | **Mean ± SEM** |  |  |
| hcmv-miR-UL112-3p | 2.62 ± 0.30 | 0.98 ± 0.08 | 2.67 | < 0.0001 |
| hcmv-miR-UL22a-5p | 0.64 ± 0.10 | 0.32 ± 0.04 | 2.00 | 0.005 |
| hcmv-miR-UL148d | 24.02 ± 4.81 | 11.40 ± 2.04 | 2.11 | 0.014 |
| hcmv-miR-UL36-5p | 7.63 ± 1.10 | 2.39 ± 0.22 | 3.19 | < 0.0001 |
| hcmv-miR-UL59 | 2.31 ± 0.28 | 1.30 ± 0.12 | 1.78 | 0.003 |

^a^ Data are presented as the Mean ± SEM

^b^ Student-t test.

**Table S3** Relative expression level of HCMV-encoded miRNAs in two types of OLP patients and normal controls ^a^

| **HCMV encoded miRNAs** | **reticular OLP (n=31)** | **erosive OLP (n=30)** | **Normal controls (n=51)** | **fold change** | | | ***P* value^b^** | | |
| --- | --- | --- | --- | --- | --- | --- | --- | --- | --- |
|  | **Mean ± SEM** | **Mean ± SEM** | **Mean ± SEM** | **reticular OLP vs Normal** | **erosive OLP vs Normal** | **reticular OLP vs erosive OLP** | **reticular OLP vs Normal** | **erosive OLP vs Normal** | **reticular OLP vs erosive OLP** |
| hcmv-miR-UL112-3p | 2.02 ± 0.25 | 3.23 ± 0.53 | 0.98 ± 0.08 | 2.05 | 3.30 | 0.63 | < 0.001 | < 0.001 | 0.054 |
| hcmv-miR-UL22a-5p | 0.48 ± 0.12 | 0.82 ± 0.16 | 0.32 ± 0.04 | 1.50 | 2.56 | 0.59 | 0.125 | < 0.001 | 0.089 |
| hcmv-miR-UL148d | 17.60 ± 5.98 | 31.03 ± 7.82 | 11.40 ± 2.04 | 1.54 | 2.72 | 0.57 | 0.25 | 0.004 | 0.178 |
| hcmv-miR-UL36-5p | 6.95 ± 1.31 | 8.34 ± 1.80 | 2.39 ± 0.22 | 2.91 | 3.49 | 0.83 | < 0.001 | < 0.001 | 0.539 |
| hcmv-miR-UL59 | 3.01 ± 0.50 | 1.63 ± 0.24 | 1.30 ± 0.12 | 2.32 | 1.25 | 1.85 | < 0.001 | 0.191 | 0.053 |

^a^ Data are presented as the Mean ± SEM

^b^ Student-t test.

**Table S4. Univariate and multivariate logistic regression analyses of plasma HCMV miRNAs for OLP.**

| **Variables** | **Univariate analysis** | | | | **Multivariate analysis** | | | |
| --- | --- | --- | --- | --- | --- | --- | --- | --- |
|  | **OR** | ***P* value** | **95% CI** | | **OR** | ***P* value** | **95% CI** | |
|  |  |  | **Lower** | **Upper** |  |  | **Lower** | **Upper** |
| hcmv-miR-UL112 | 6.044 | 0.006 | 1.659 | 22.024 | 6.000 | 0.006 | 1.621 | 22.208 |
| hcmv-miR-UL22a-5p | 2.370 | 0.221 | 0.595 | 9.447 | ‐ | 0.747 | ‐ | ‐ |
| hcmv-miR-UL148d | 3.077 | 0.102 | 0.799 | 11.852 | ‐ | 0.501 | ‐ | ‐ |
| hcmv-miR-UL36-5p | 5.565 | 0.010 | 1.520 | 20.374 | ‐ | 0.073 | ‐ | ‐ |
| hcmv-miR-UL59 | 5.565 | 0.010 | 1.520 | 20.374 | ‐ | 0.073 | ‐ | ‐ |

**Table S5. Targets of HCMV-encoded miRNAs.**

| **Target** | **miRNA** | **Target gene** | **Description** | **Validated**  **or not** |
| --- | --- | --- | --- | --- |
| HCMV | hcmv-miR-UL112 | IE72 | immediate-early protein 1 | YES |
|  |  | UL120/121 | involved in viral replication | YES |
|  |  | UL112/113 | involved in viral replication | YES |
|  |  | UL114 | uracil DNA glycosylase | YES |
|  |  | UL102 | involved in DNA replication | NO |
|  |  | UL56 | involved in DNA encapsidation | NO |
|  | hcmv-miR-UL148d | UL37 | encoding regulatory proteins at immediate early | NO |
|  | hcmv-miR-UL36-5p | UL138 | latency-associated gene | YES |
|  |  | UL54 | involved in viral replication | NO |
|  | hcmv-miR-UL59 | US2 | involved in immune regulation | NO |
| Human | hcmv-miR-UL112 | MICB | a member of NKG2Dligands | YES |
|  |  | VAMP3 | contributing to the release of proinﬂammatory cytokines TNF-a and IL-6 | YES |
|  |  | SNAP23 |  | YES |
|  |  | RAB5C |  | YES |
|  |  | RAB11A |  | YES |
|  |  | IL-32 | regulated on activation | YES |
|  |  | MICA | a member of NKG2D ligands | NO |
|  | hcmv-miR-UL22a-5p | BMPR2 | bone morphogenetic protein receptor type II | YES |
|  |  | C-MYC | a transcriptional regulator | YES |
|  | hcmv-miR-UL148d | IEX-1 | apoptotic-related protein | YES |
|  |  | RANTES | regulated on activation | YES |
|  |  | SOCS5 | suppressor of cytokine signaling 5 | NO |
|  |  | ST14 | tumor suppressor | NO |
|  |  | TP53BP1 | inhibiting cell growth, invasion and metastasis | NO |
|  | hcmv-miR-UL36-5p | Skp2 | oncoprotein regulating tumor suppressor proteins | NO |
|  | hcmv-miR-UL59 | RASSF2 | tumor suppressors contributing to brain metastases | NO |
|  |  | ULBP1 | a member of NKG2D ligands | NO |

**Reference**

1. Murphy E, Vanicek J, Robins H, Shenk T, Levine AJ: **Suppression of immediate-early viral gene expression by herpesvirus-coded microRNAs: implications for latency.** *Proc Natl Acad Sci U S A* 2008, **105:**5453-5458.

2. Grey F, Meyers H, White EA, Spector DH, Nelson J: **A human cytomegalovirus-encoded microRNA regulates expression of multiple viral genes involved in replication.** *PLoS Pathog* 2007, **3:**e163.

3. Stern-Ginossar N, Saleh N, Goldberg MD, Prichard M, Wolf DG, Mandelboim O: **Analysis of human cytomegalovirus-encoded microRNA activity during infection.** *J Virol* 2009, **83:**10684-10693.

4. Huang Y, Qi Y, Ma Y, He R, Ji Y, Sun Z, Ruan Q: **Down-regulation of human cytomegalovirus UL138, a novel latency-associated determinant, by hcmv-miR-UL36.** *J Biosci* 2013, **38:**479-485.

5. Stern-Ginossar N, Elefant N, Zimmermann A, Wolf DG, Saleh N, Biton M, Horwitz E, Prokocimer Z, Prichard M, Hahn G, et al: **Host immune system gene targeting by a viral miRNA.** *Science* 2007, **317:**376-381.

6. Hook LM, Grey F, Grabski R, Tirabassi R, Doyle T, Hancock M, Landais I, Jeng S, McWeeney S, Britt W, Nelson JA: **Cytomegalovirus miRNAs target secretory pathway genes to facilitate formation of the virion assembly compartment and reduce cytokine secretion.** *Cell Host Microbe* 2014, **15:**363-373.

7. Lisboa LF, Egli A, O'Shea D, Asberg A, Hartmann A, Rollag H, Pang XL, Tyrrell DL, Kumar D, Humar A: **Hcmv-miR-UL22A-5p: A Biomarker in Transplantation With Broad Impact on Host Gene Expression and Potential Immunological Implications.** *Am J Transplant* 2015, **15:**1893-1902.

8. Wang YP, Qi Y, Huang YJ, Qi ML, Ma YP, He R, Ji YH, Sun ZR, Ruan Q: **Identification of immediate early gene X-1 as a cellular target gene of hcmv-mir-UL148D.** *Int J Mol Med* 2013, **31:**959-966.

9. Kim Y, Lee S, Kim S, Kim D, Ahn JH, Ahn K: **Human cytomegalovirus clinical strain-specific microRNA miR-UL148D targets the human chemokine RANTES during infection.** *PLoS Pathog* 2012, **8:**e1002577.
